# Open triple fusion versus TNC arthrodesis in the treatment of Mueller-Weiss disease

**DOI:** 10.1186/s13018-017-0513-3

**Published:** 2017-01-19

**Authors:** Hongtao Zhang, Junkun Li, Yusen Qiao, Jia Yu, Yu Cheng, Yan Liu, Chao Gao, Jiaxin Li

**Affiliations:** 1 Department of Orthopedics, The First Affiliated Hospital of Soochow University, Suzhou, 215006 China; 2 Orthopedic Institute, Soochow University, 708 Renmin Rd, Suzhou, Jiangsu 215007 China; 3 Department of Mathematics, University of Waterloo, Waterloo, N2L 3G1 Canada

**Keywords:** Mueller-Weiss disease, Triple fusion, Arthrodesis, Navicular

## Abstract

**Background:**

Mueller-Weiss disease is a rarely diagnosed deformity where the navicular bone undergoes spontaneous osteonecrosis in adults. Until now, there is no widely accepted operative treatment for this unusual disease. We aimed to compare clinical and radiological outcomes between the open triple fusion and talonavicular-cuneiform arthrodesis for Mueller-Weiss disease of stage 4.

**Methods:**

During the period from February 2012 to June 2016, 10 patients (11 feet) suffering from Mueller-Weiss disease of stage 4 were treated by the same senior surgeon. Among them, 5 patients (5 feet) were treated with open triple fusion and 5 patients (6 feet) were treated with talonavicular-cuneiform arthrodesis. Clinical outcomes were evaluated by American Orthopaedic Foot and Ankle Society (AOFAS) ankle-hindfoot score. Radiological results were assessed based on the X-ray and CT. Postoperative complications were also recorded.

**Results:**

There were no significant differences in AOFAS score between the two groups (*p* = 0.1 > 0.05). For the open triple fusion, the average AOFAS ankle-hindfoot score improved from 30.2 ± 3.27 preoperatively to 79 ± 3.81 at the last follow-up (*p* = 0.008). And for the talonavicular-cuneiform (TNC) arthrodesis, the average AOFAS ankle-hindfoot score improved from 33.2 ± 5.63 preoperatively to 86.2 ± 3.49 at the last follow-up (*p* = 0.007).

**Conclusions:**

Both triple fusion and TNC arthrodesis are reasonable methods for the treatment of Mueller-Weiss disease if properly used. It is crucial to use radiological assessment to evaluate the involved joints preoperatively and then chose the appropriate method to treat different patients.

## Background

**Fig. 1:**
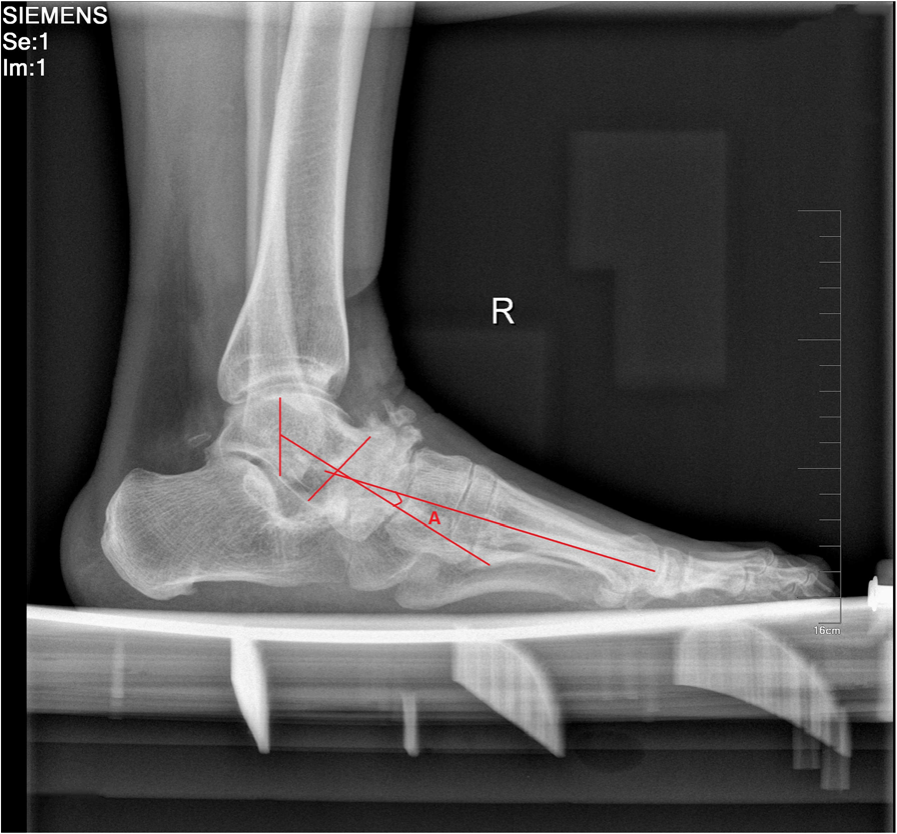
Lateral weightbearing radiograph showing severe sclerosis of the navicular and representation of stage 4 according to the Maceira classification. *Angle A* denotes the Meary-Tomeno’s angle

Mueller-Weiss disease (MWD) is a complicated idiopathic foot condition, presenting as a chronic midfoot pain with deformity of the tarsal navicular in adults. True prevalence and incidence of this disease is still unknown so far. MWD is more frequently bilateral and commonly present in women from 40 to 60 years old [1, 2]. The mean age at diagnosis in one series was 47.6 years (range 13–91 years) [3]. MWD possibly occurs more common in Europe than America [3], suggesting a possible environmental and nutritional link to MWD. But a recent study by Doyle did not identify any environmental or social factor as predisposing factor [4]. Many possible causes have been proposed, including primary osteonecrosis, traumatic or biomechanical factors, peri-navicular osteoarthritis, congenital malformation, and abnormal evolution of Kohler’s disease, but the most generally accepted causes are delayed ossification of the navicular and an abnormal force distribution pattern [3]. The reason of abnormal force distribution at the medial heel in foot of MWD may be hindfoot varus deformity. Most of the patients typically complain of chronic midfoot pain, swelling, and tenderness on the dorsal and medial midfoot. Deformity such as flatfoot with varus heel on the dorsal side is often observed. Pes planovarus deformity in its advanced stages is even considered to be the hallmark of MWD [5]. Plain weightbearing radiographs and clinical examination are usually sufficient to diagnose the disease. Typical radiological findings of the navicular bone in MWD are a loss of volume with increased radiodensity, a comma-like shaped configuration due to compression, a subsequent medial or dorsal protrusion, and a fragmentation of the navicular bone [1, 6]. Maceira et al. [3] described five radiographic stages of MWD according to the sagittal plane deformity of the navicular bone and the orientation of the intersection of the talar and first metatarsal axes (Meary-Tomeno’s angle) (Fig. 1).

For the treatment of MWD, many researches suggest initial non-surgical treatment, range from 2 [7] to 60 [8] months. Conservative management is composed of insoles, orthoses, decreased physical activity, non-weightbearing cast immobilization, and/or nonsteroidal anti-inflammatory medication [7–12], but it often fails when using immobilization by orthoses and anti-inflammatory medications [9, 13]. Indeed, surgery is required in a large number of cases. Operative treatment should be considered when conservative treatment failed to relieve the symptom [7]. Several operative techniques have been proposed for midfoot pain relief and deformity correction: internal fixation of navicular bone, simple excision of the dorsolateral fragment of the navicular with bone graft [10, 14], percutaneous drilling decompression [12], isolated talonavicular arthrodesis [10, 15, 16], talonavicular-cuneiform (TNC) arthrodesis [7], and double fusion or triple arthrodesis [8]. However, it remains uncertain that which kind of treatment is the best method to treat which type of MWD. Thus, we were interested in determining whether the open triple fusion and TNC arthrodesis would provide comparable clinical outcomes. The purpose of this study was to evaluate clinical and radiological results after TNC arthrodesis and open triple fusion with MWD, classified as stage 4 by Maceira [3]. We hypothesized that there exists a difference in surgical outcomes of two procedures.

## Methods

We reviewed the records of 11 feet from 10 patients (9 women and 1 man) with MWD who received surgery at the First Affiliated Hospital of Soochow University from February 2012 to June 2016. The patients fulfilled the inclusion criteria and were treated with either open modified triple fusion or TNC arthrodesis. The Ethics Committee of the Hospital had approved the study. Informed consent was obtained from the patient or from his or her relatives if the patient was incapable to give consent. Patients who were admitted to the hospital with a MWD during the study period were considered eligible for the study.

**Fig. 2:**
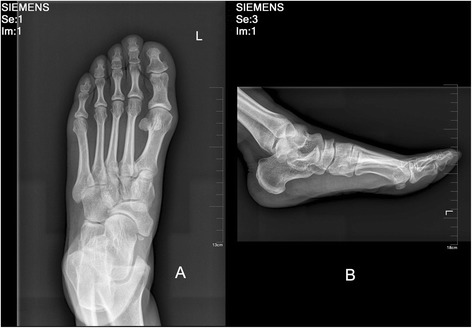
**a** Anteroposterior and **b** lateral radiograph of the foot indicate a Mueller-Weiss disease preoperatively

The diagnosis was established based on one’s medical history, clinical examination, and radiological evaluation. Clinically, patients exhibited pain and tenderness at the dorsomedial aspect of the midfoot. Navicular necrosis was shown in radiologic evaluation, including plain radiographs, CT, and MRI (Fig. 2). Inclusion criteria were (1) attained full legal age, (2) presented with severe midtarsal pain, (3) a minimum of 2 months of conservative treatment using insole and physiotherapy had failed, and (4) arthritis of the TN joint was present preoperatively in at least one of the different radiographs taken. Patients with Kohler disease, Charcot arthropathy of the midfoot, navicular stress injury (response or fracture), or a navicular traumatic fracture were excluded. The cases were graded by lateral weightbearing radiograph according to the Maceira staging system [3]. Following these guidelines, 10 patients (11 feet) were included in this study and randomly allocated into group A (open triple fusion) and group B (talonavicular-cuneiform arthrodesis). All surgeries were performed by the same senior surgeon.

Outcome measures included the ankle-hindfoot scale of the American Orthopaedic Foot and Ankle Society (AOFAS) [17] and radiographic assessment. A fusion was deemed successful if the fusion site became painless on both weightbearing and manipulation and if radiographs demonstrated trabeculation across the fusion site.

Patients’ medical records were reviewed and then collected. The collected demographic and clinical data includes patient age, sex, side of the involved foot, preoperative clinical and radiological evaluation, operating procedure, postoperative clinical and radiological evaluation, complications, and follow-up clinical and radiological outcome.

### Surgical techniques

#### Group A

**Fig. 3:**
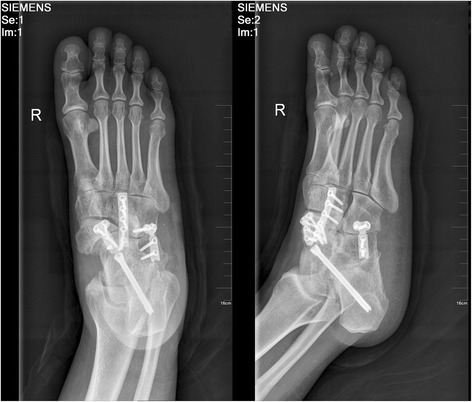
Postoperative radiograph with open triple fusion

All procedures were performed in the supine position after inducing satisfactory spinal anesthesia with a thigh tourniquet. The subtalar and calcaneocuboid joints were exposed through a lateral incision (oblique sinus tarsi incision) in order to take care and to protect the peroneal tendons, sural nerve, and superficial peroneal nerve. The talonavicular joint was approached through a longitudinal medial incision (starts from the medial malleolus and extends to the NC joint). Once all three joints (if NC joint also involved, then four joints) had been exposed, their osteophytes and diseased articular cartilage were removed to expose the subchondral bone. Then, the residual cartilage and sclerotic bone of the involved bone was cut by an osteotome to form a dorsal broad and plantar narrow bony bed for the bone block at the talonavicular joint and debrided with a curette and high-speed burr with chilled 6 °C (43 °F) saline until healthy, rough bone surface was prepared and the surface were drilled to a faviform texture to facilitate fusion. The tourniquet was deflated to check blood perfusion and reinflated afterwards. A tricortical autogenous graft of the same size and shape was then obtained from the iliac crest and inserted in the bed. Arthrodesis of all three joints (or even four joints) was performed with applicable screws and plate to stabilize the foot. The rest of the cancellous bone was used to fill the defects, and then the wound is irrigated, closed, and dressed. Fluorescence was used during the whole surgery in order to ensure that the placement of the hardware was optimal (Fig. 3).

#### Group B

**Fig. 4:**
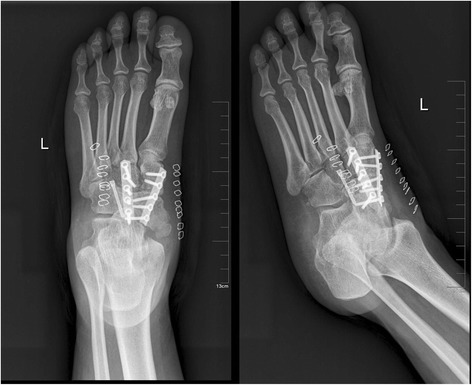
Postoperative radiograph with talonavicular-cuneiform arthrodesis

All procedures were performed in the supine position after inducing satisfactory spinal anesthesia with a thigh tourniquet. The navicular, cuneiforms, and the head of the talus were exposed through a longitudinal dorsal incision (about 6 cm) between the anterior tibial tendon and the extensor hallucis longus. Then the capsules of the talonavicular joint (TNJ) and naviculocuneiform joint (NCJ) were incised longitudinally. A lamina spreader was used to distract the soft tissue and expose the TNJ and NCJ joints. If a bony prominence was found, it was excised by an osteotome. The residual cartilage and sclerotic bone of the involved bone was cut using an osteotome in order to form a dorsal broad and plantar narrow bony bed for the bone block and was debrided using a curette and high-speed burr with chilled 6 °C (43 °F) saline until healthy, rough bone surface was well-prepared and the surface was drilled to a faviform texture for fusion. The tourniquet was deflated to check blood perfusion and then reinflated. After removing the cartilage of the talonavicular-cuneiform articular surface, a tricortical autogenous graft of the same size and shape was obtained from the iliac crest and inserted into the bed. Arthrodesis of the TNJ and NCJ were performed with appropriate screws and plate to stabilize the bone block between the talus and cuneiforms. The rest of the cancellous bone was used to fill the defects, and then the wound was irrigated, closed, and dressed. Fluorescence was used during the whole surgery, in order to ensure the placement of the hardware was optimal (Fig. 4).

### Postoperative managements

Patients were immobilized in a plaster cast for 6 weeks. Six weeks after the surgery, the patients began to walk with help of a partially weightbearing crutch. Three months after the surgery, the patients may be allowed to walk with full weightbearing depending on bone fusion by radiograph.

### Statistical analysis

Statistical analysis was performed using SPSS statistical package, version 13 (SPSS Inc., Chicago, IL, USA) for Windows. Student’s *t* test was used to compare the means of difference of AOFAS score between two operative methods. Paired Student’s *t* test was used to compare the means of preoperative and postoperative AOFAS score for each method. A *p* value <0.05 was considered statistically significant.

## Results

There are 12 patients fulfilling the inclusion criteria of this study. One of them was unable to be reached and another refused to participate. Among the remaining 10 patients, 5 of them (5 feet) had undergone open triple fusion (group A) and 5 (6 feet) talonavicular-cuneiform arthrodesis (group B). One patient had bilateral involvement in group B.

The basic information about the patients was summarized in Table 1. The mean age of the patients at the time of surgery was 48.1 (range 22 to 59) years. The mean duration between the onset of syndrome and the operation was 3.65 (range 0.5 to 10) years. One patient (10%) was male, and six (90%) were female. Six (55%) operated feet were on the left side and five (45%) were on the right. Pes planus was present in 10 (91%) feet. Pes cavus was present in one (9%) foot.

**Table 1:** Basic information of total collective, triple fusion, and TNC arthrodesis

Characteristic	Total	Triple fusion	TNC arthrodesis
Feet	11	5	6
Mean follow-up (months)	7.5 (range 1 to 28)	6.8 (range 1 to 12)	8.2 (range 2 to 28)
Mean age (years)	48.1 (range 22 to 59)	52.8 (range 47 to 59)	43.4 (range 22 to 58)
Sex			
Female	9 (90%)	5 (100%)	4 (80%)
Male	1 (10%)	0	1 (20%)
Operated side			
Left	6 (55%)	3 (60%)	3 (50%)
Right	5 (45%)	2 (40%)	3
Duration of symptoms (years)	3.65 (range 0.5 to 10)	4.7 (range 0.5 to 10)	2.6 (range 0.5 to 6)
Deformity			
Pes planus	10 (90%)	5 (100%)	5 (83%)
Pes cavus	1 (10%)	0	1 (17%)
Hindfoot varus	0	0	0
AOFAS score (points)			
Preoperative	31.7 (range 25 to 38)	30.2 (range 25 to 33)	33.2 (range 25 to 38)
Last follow-up	82.6 (range 75 to 90)	79 (range 75 to 85)	86.2 (range 82 to 90)

The mean time duration between operation and follow-up was 7.5 (range 1 to 28) months. For group A, the average AOFAS ankle-hindfoot score was improved from 30.2 ± 3.27 (preoperative score) to 79 ± 3.81 in the last follow-up (*p* = 0.008). And for group B, the average AOFAS ankle-hindfoot score was improved from 33.2 ± 5.63 (preoperative score) to 86.2 ± 3.49 in the last follow-up (*p* = 0.007). In addition, there was no significant difference in AOFAS score between the two groups (*p* = 0.1).

The results of radiological assessment are presented in Table 2. In regard to the preoperative radiographic evaluation of the navicular, the AP view showed that there was compression in 1 foot (1 in group B), comma shape in 5 feet (2 in group A and 3 in group B), and collapse in 5 feet (3 in group A and 2 in group B); the lateral view revealed that there was compression in 2 feet and fragment in 9 feet. There was osteoarthritis observed in 11 TNJ, 11 NCJ, 4 subtalar joints, and 3 calcaneocuboid joints. CT scan demonstrated a cystic lesion in the head of the talus within each of the 11 feet. According to the Maceira staging system [3], all feet were at stage 4. The mean time duration of radiologic union was 13(range 11 to 16) weeks.

**Table 2:** Radiological evaluation of total collective, triple fusion, and TNC arthrodesis

Variable	Total	Triple fusion	TNC arthrodesis
X-ray evaluation			
AP view			
Compression	1 (10%)	0	1 (17%)
Comma shape	5 (45%)	2 (40%)	3 (50%)
Collapse	5 (45%)	3 (60%)	2 (33%)
Lateral view			
Compression	2 (18%)	0	2 (40%)
Fragment	9 (82%)	5 (100%)	4 (80%)
Osteoarthritis			
Ankle joint	0	0	0
TNJ	11 (100%)	5 (100%)	6 (100%)
NCJ	7 (63%)	1 (20%)	6 (100%)
Subtalar joint	4 (36%)	4 (80%)	0
CCJ	1 (9%)	1 (20%)	0
CT evaluation			
Cystic lesion of talar head	11 (100%)	5 (100%)	6 (100%)
Time to radiological union (weeks)	13 (range 11 to 16)	15 (range 14 to 16)	12 (range 11 to 14)

## Discussion

The purpose of this study was to evaluate the clinical and radiological results of using open triple fusion and TNC arthrodesis to treat MWD. The main finding of this investigation was that open modified triple fusion and TNC arthrodesis are equally effective for the relief of the symptoms of MWD. There was no significant difference between the two operative methods according to preoperative and postoperative AOFAS score. It is crucial to use radiological assessment to evaluate the involved joints preoperatively and then choose the appropriate method to treat each individual patient.

Mueller-Weiss disease refers to spontaneous osteonecrosis of the navicular in adults, and it occurs more often in women than in men by a ratio of 6:4 [1, 12]. Most patients complained about long-standing midfoot pain on the dorsum of foot. The typical foot deformity can be tolerated for years [3]. The results of this investigation are similar to the findings of former studies. In our study, all patients had reported unbearable chronic pain on the foot, which made them decide to undergo an operation. There were more women (90%) than men (10%), and the mean age of the patients was 48.1 years old. The mean time duration of radiologic union was 13 weeks. The patients included in this paper all experienced a 100% fusion rate except a patient who had his postoperative follow-up only 8 weeks. Pes planus was shown in most (90%) feet in this study, which is similar to previous reports. Because fragmentation affects the lateral part of the navicular, the talar head protrudes outwards, resulting in pes planovarus [3, 7]. Paradoxical pes planusvarus suggests an advanced stage of disease and is usually associated with prominent calcaneum posteriorly due to relative advancement of the tibia in relation to the tarsal joints. However, there is one patient (10%) having high-arch foot in our study, which may be attributed to the fact that the patient does not often walk and thereby shows less weightbearing and slower progress of MWD. Previous paper also shows that patients with MWD can demonstrate a normal, high-, or low-arched foot with rearfoot varus [3]. A prominent navicular tuberosity can give a false impression of valgus hindfoot [3]. Maceira et al. [3] found that the decreased pressure of the forefoot in MWD could lead to a low incidence of hallux valgus. This phenomenon also appeared in all of our patients. There was no patient suffering from MWD combined with hallux valgus in our study.

**Fig. 5:**
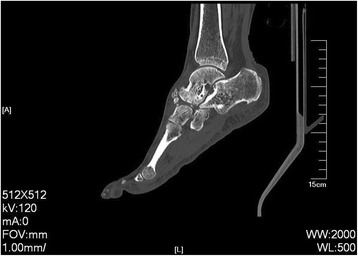
CT scan showing degenerative changes in the subtalar joint

Until now, there is no widely accepted surgical treatment for this uncommon disease, though some treatments were recommended to correct the deformity and release the pain. Internal fixation of navicular bone is considered to be a good choice to treat some acute fractures of the navicular. But in MWD, the lack of bone stock makes it very difficult to perform the operation ideally since the bone is necrotic. Maceira et al. [7] reported unsatisfactory results of internal fixation in the presence of arthritic changes around the navicular bones. Percutaneous decompression of the navicular was suitable for early stages of MWD. However, in practice, most of the patients have established arthritic changes of mid- and hindfoot deformity by the time the condition is diagnosed, and at this stage, core decompression is unlikely to be helpful [12]. Isolated talonavicular arthrodesis usually fails to resolve the incongruence of naviculocuneiform joint and carries a high risk of pseudarthrosis. Triple fusion results in better consolidation but does not address naviculocuneiform arthrosis-related symptoms. TNC arthrodesis may cause pseudarthrosis at one of the joints, and if pseudarthrosis develops, the cortical graft may be fractured or luxated from bed and reoperation is usually necessary [7]. Fernandez et al. [7] revealed that triple fusion results in better consolidation than isolated talonavicular arthrodesis. Triple fusion can provide medial and lateral stability. Maceira et al. [3] maintained using TNC arthrodesis, insisting that triple fusion did not address degeneration at the naviculocuneiform joint and the subtalar fusion was also unnecessary. However, Lui et al. [8] showed significant incidence of calcaneocuboid degeneration, which cannot be detected by preoperative X-rays. Coughlin et al. [18] also suggested that adding a calcaneocuboid arthrodesis instead of an isolated talonavicular arthrodesis is necessary because of the potential pain in the calcaneocuboid joint. As Lu et al. [15] recommended, in advanced MWD with marked deformity and adjacent joints arthritic change, the surgical treatment should be triple fusion, TNC arthrodesis, or double fusion. Mayich et al. [19] suggested that if stage 4 disease is present, additional subtalar fusion is mostly required. Likewise, we found most patients have the degenerative changes in the subtalar joints by CT scan in our triple fusion group (Fig. 5). Furthermore, we also found the osteoarthritis of calcaneocuboid in some patients during the triple fusion operation and we thus performed the triple fusion on these patients. We also found that NC joint was involved in the triple fusion group, so we added a NC joint fusion to the triple fusion to relieve pain. As Doyle [4] reported, in advanced grades of MWD involving the naviculocuneiform joint, triple fusion can be extended to include the NC joint. But in some cases, patients had difficulties on uneven ground because of the range of hindfoot motion. We tentatively advocate that the triple fusion for MWD is suitable to treat advanced-stage patients who are relatively old (no more requirements on the range of hindfoot motion) and have degenerative changes observed in the CC and/or subtalar joints (evaluate from the radiological assessment or intra-operation).

Talonavicular-cuneiform arthrodesis was recently reported with favorable reviews. Most of them used bone graft either autograft or allograft. Retana et al. [7] showed that TNC arthrodesis using trapezoid autologous graft can achieve arthrodesis and regular medial arch length. Cao et al. [20] has reported TNC arthrodesis with a reverse V-shaped osteotomy through the talonavicular joint. In our study, TNC arthrodesis group used shorter union time than the triple fusion group. This phenomenon may happen because patients in TNC fusion group were generally younger patients and had fewer joints involved. Although there was no osteoarthritis in the subtalar or calcaneocuboid joint by X-ray or CT preoperatively, the patient should pay additional attention to these two joints postoperatively to avoid adjacent osteoarthritis changes [21].

Limitations of the present study include the small number of sample cases and that the fusion was certified by plain radiographs instead of CT scans. Because of the short length of follow-up time, we were unable to evaluate the incidence or extent of subsequent periarticular degenerative changes by these two techniques. Long-term follow-up may lead to additional investigation about the degeneration of adjacent joints. We would cooperate with some clinical centers to collect enough sample cases so that we can analyze the results thoroughly.

## Conclusions

Both triple fusion and TNC arthrodesis are reasonable methods to treat Mueller-Weiss disease. If the subtalar and/or calcaneocuboid joints are involve, triple fusion may be an effective and reliable operative option. It is crucial to use radiological assessment to evaluate the involved joints preoperatively and then chose the appropriate method to treat each patient specifically.

## Abbreviations

MWD: Mueller-Weiss disease
TNC: Talonavicular-cuneiform
